# The Accuracy of Surface Analyses

**DOI:** 10.6028/jres.093.087

**Published:** 1988-06-01

**Authors:** C. J. Powell

**Affiliations:** Surface Science Division, National Bureau of Standards, Gaithersburg, MD 20899

## 1. Introduction

Surface analysis is being increasingly used for a great variety of scientific and technological applications [1,2]. The composition of the outermost or several outermost atomic layers of a selected region on a specimen surface is frequently measured. It is also possible to measure the compositional variations with position on the surface and, in conjunction with removal of surface layers by ion bombardment, to obtain composition-versus-depth information. Such analytical information is often critical for the solution of a wide range of problems.

While many of the applications of surface analysis have been successful in fulfilling their intended purposes, there have been many difficulties in achieving high accuracy, as will be described below. The precision (repeatability) of many surface analyses is generally satisfactory for most purposes (often 1% and sometimes better). The precision depends largely on the extent of uniformity of different regions of a specimen surface (if replicate measurements are made), instrumental stability, the statistical quality (signal-to-noise ratio) of the acquired data, and the extent of specimen change during data acquisition. Specimen surfaces may change in composition during measurements due to decomposition or diffusion induced by the incident radiation; the composition may also change due to adsorption of molecules from the ambient vacuum, to segregation from the bulk (particularly if the surface is heated), or to differential sputtering effects on ion-bombarded surfaces. Thus, gains in precision obtained by increasing the measurement time may be offset by a loss of analysis accuracy if the surface composition changes. A similar problem arises with attempts to measure the composition of small regions with highly focused incident beams; as the beam diameter is decreased, the beam current is reduced and it can be counterproductive to increase the signal (sensitivity) by increasing the measurement time on account of beam-induced damage to the specimen.

A brief summary is given here of the many factors that can limit the accuracy of surface analyses made with the techniques in common use (Auger-electron spectroscopy (AES), x-ray photoelectron spectroscopy (XPS), and secondary-ion mass spectroscopy (SIMS)). Additional details are given elsewhere [2,3].

## 2. Factors Affecting the Accuracy of Surface Analyses

It is unfortunately not often possible for the average analyst to make credible claims for the accuracies of surface analyses made on practical materials. There are many factors which both complicate any analysis and make statements of systematic error (bias) either impossible or extremely difficult. Such factors can be classified as follows.

### 2.1 Specimen Complexity

**Figure 1:**
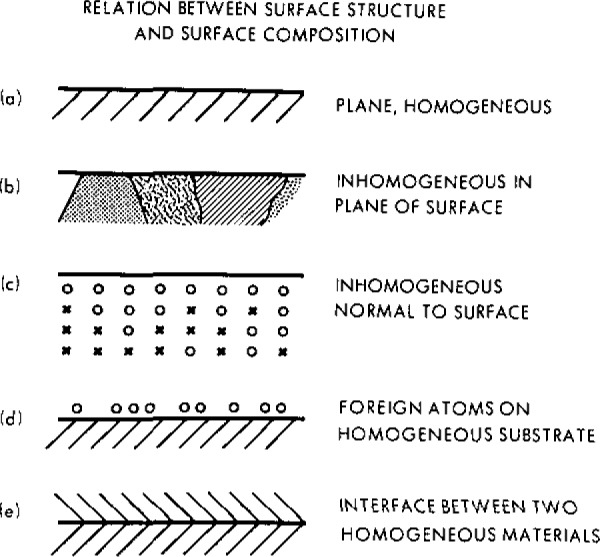
Idealized surface morphologies: (a) plane homogeneous surface; (b) a surface with lateral inhomogeneities consisting of several different surface phases; (c) a surface with depth inhomogeneities (the circles and the crosses represent different types of atoms); (d) a surface phase consisting of a submonolayer of foreign atoms on an otherwise homogeneous surface; and (e) an interface between two homogeneous bulk phases [3].

It is often implicitly assumed that a specimen is compositionally homogeneous over the volume probed in the measurement. Many specimens, however, are inhomogeneous, as illustrated schematically in figure 1. Since surface-analysis techniques are sensitive to the outermost few atomic layers, it is necessary to characterize the variation of composition with depth on an atomic scale in order to ensure that observed spectral intensities can be meaningfully converted to elemental concentrations.

The surfaces of practical specimens are unlikely to be smooth, either on a microscopic or a macroscopic scale. Details of the surface topography (as well as local defects) can affect particle transport and the relative intensities of different spectral features. Similarly, complex specimen geometries (e.g., impurity particles, the presence of islands in deposited films, or thin lines on microelectronic devices) may require an appropriate model calculation in order to drive a composition from observed signals.

If the specimen material is a single-crystal (or an epitaxial film on a single-crystal substrate), the angular distributions of emitted electrons (in AES or XPS) or ions (in SIMS) can be anisotropic. Changes of observed intensities of ~30% in AES have been found when the incident beam was deflected from one grain to another in a polycrystalline specimen or when the angle of incidence was varied [4,5]. Analyses of such transport anisotropies are complex.

### 2.2 Instrument Performance

**Figure 2:**
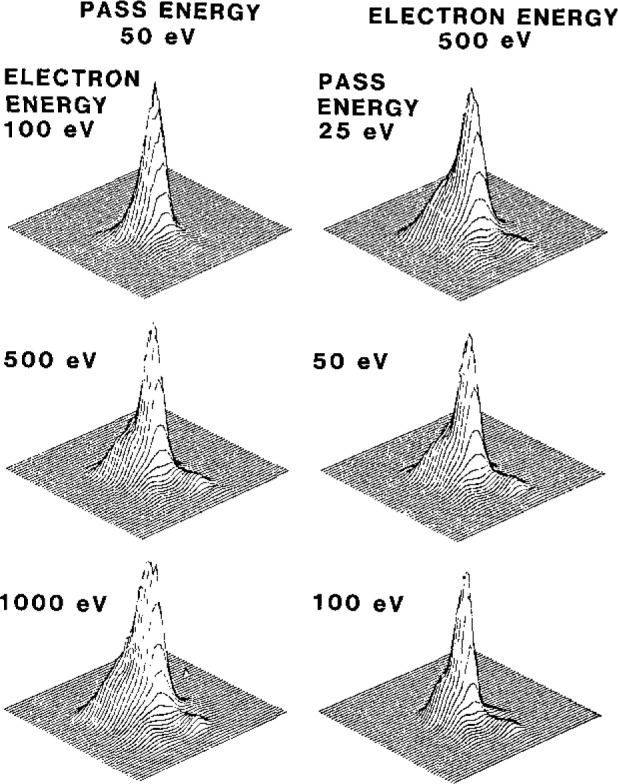
Images recorded with a double-pass cylindrical-mirror analyzer to show the specimen areas contributing to the detected signal for the indicated conditions of analyzer pass energy and electron kinetic energy. The horizontal (bottom left to right) distance is 13 mm and the vertical distance is 15 mm.[8].

It is clearly important that the intensity scale of an instrument be linear and change in known ways if instrumental parameters are varied. An early XPS laboratory intercomparison showed variations in intensity ratios for the major peaks in each of two pure metals of up to a factor of 10, a result attributed in part to erratic instrument performance [6]. A later XPS intercomparison showed an order of magnitude improvement [7] that was presumably due to instrumental advances. An instrument should also “view” a well-defined region of the specimen, particularly if there are compositional inhomogeneities in the plane of the specimen. Figure 2 nevertheless indicates how an XPS analyzer can view different specimen areas depending on the selected operating parameters [8]; such variations in observed specimen area need to be known when comparing intensities at different electron energies.

Modern instruments are typically supplied with computer systems and proprietary software developed by the manufacturer. Instrument automation has many advantages, but the software may have limitations that can cause unsuspected inaccuracies in an analysis [2]. An example of such a limitation is the use of oversimplified (yet convenient) methods for background determination and thus for intensity measurement in AES and XPS. It is usually difficult for an analyst to make desired changes to software.

### 2.3 Lack of Needed Reference Data and Reference Materials

Spectral lineshapes (in AES and XPS) and intensities (in AES, XPS, and SIMS) for a particular element often depend on the matrix in which the element is found. Individual parameters important in observed matrix effects are electron attenuation lengths and inelastic mean-free paths (AES, XPS), back-scattering factors (AES), ion neutralization cross sections (SIMS), and sputtering rates (SIMS). The magnitudes of the matrix effects on these individual parameters and on spectral intensities (sensitivity factors) need to be either documented or calculated from theory. A review of data compilations needed in AES and XPS has been published by Seah [9].

**Figure 3:**
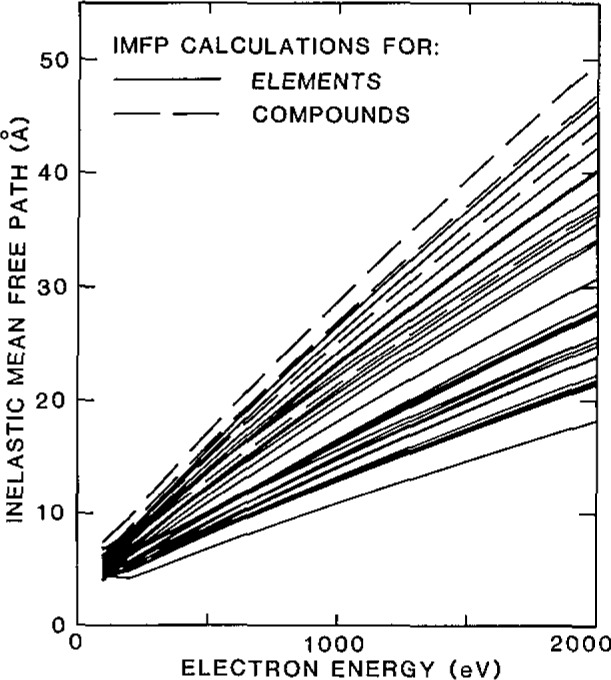
Plots of calculated electron inelastic mean-free paths versus electron energy for 27 elements (solid lines) and 4 compounds (dashed lines) [10].

One of the most important matrix corrections in AES and XPS involves the electron inelastic mean-free path (IMFP). Accurate IMFP measurements are difficult to make and there has been considerable uncertainty over the IMFP dependence on electron energy and material. Figure 3 shows results of new IMFP calculations for 27 elements and 4 compounds and indicates the IMFP range that can be encountered [10].

Reference materials are needed for calibration of instrumental energy (AES, XPS) and intensity scales, demonstration of detection sensitivity, calibration of depth scales in sputter-depth profiling (SDP), and optimization of profiling parameters in SDP. A recent article gives details of the limited number of reference materials now available for these purposes [11].

## 3. Summary

The accuracy of surface analyses generally depends on the particular sample, the analysis technique, the analytical procedure, and the particular measuring instrument. Much useful progress has been made in recent years in developing improved methodologies and in providing needed reference data, reference materials, and reference procedures [12]. Surface analysts still need additional assistance in these areas to ensure that analyses of known accuracy can be made routinely.
